# Journal of ovarian research reviewer acknowledgement 2014

**DOI:** 10.1186/s13048-015-0157-z

**Published:** 2015-07-01

**Authors:** Sham S Kakar, Stefano Palomba, Benjamin K Tsang, David T Curiel

**Affiliations:** Department of Physiology and Biophysics, University of Louisville, Louisville, KY 40202 USA; Department of Obstetrics, Gynecology and Pediatrics, Sterility Centre P. Bertocchi, Obstetrics and Gynecology Unit, A.O. S. Maria Nuova, IRCCS, Reggio Emilia and University of Modena, Reggio Emilia, Italy; Department of Obstetrics & Gynaecology, University of Ottawa and the Chronic Disease Program, Ottawa Hospital Research Institute, Ottawa, K1H 8 L6 Canada; Cancer Biology Division, School of Medicine, Washington University in St. Louis, St. Louis, MO 63108 USA

## Abstract

The editors of *Journal of Ovarian Research* would like to thank all of our reviewers who have contributed to the journal in Volume 7 (2014).

**Abban-Mete Gülcin**

Turkey

**Alviggi Carlo**

Italy

**Amorim Christiani**

Belgium

**Angioli Roberto**

Italy

**Anschau Fernando**

Brazil

**Archer Gary**

United States of America

**Argenta Peter**

United States of America

**Arisan Elif Damla**

Turkey

**Aydin Yusuf**

Turkey

**Baka Stavroula**

Greece

**Bao Shisan**

Australia

**Barbolina Maria**

United States of America

**Barua Animesh**

United States of America

**Bashiri Asher**

Israel

**Bast Robert**

United States of America

**Bellis Susan**

United States of America

**Bermudez Yira**

United States of America

**Bevilacqua Estela**

Brazil

**Bhartiya Deepa**

India

**Bidzinski Mariusz**

Poland

**Bosenberg Marcus**

Brazil

**Branchini Gisele**

Brazil

**Bukovsky Antonin**

Czech Republic

**Büldt Georg**

Germany

**Buxton David**

United States of America

**Cannon Mark**

United States of America

**Cao Cong**

China

**Capodanno Francesco**

Italy

**Catteau-Jonard Sophie**

France

**Cecconi Sandra**

Italy

**Cedar Marcelle I.**

United States of America

**Cheema Birinder**

Australia

**Chen Shuo**

China

**Chen Mei-jou**

Taiwan

**Chian Ri-Cheng**

Canada

**Christenson Lane**

United States of America

**Christman Gregory**

United States of America

**Chudecka-Glaz Anita**

Poland

**Cognasse Fabrice**

France

**Constantinou Pamela**

United States of America

**Corleta Helena**

Brazil

**Cosar Emine**

Turkey

**Dahan Michael**

Canada

**Dane Banu**

Turkey

**Davis John S.**

United States of America

**De Leo Vincenzo**

Italy

**DiFeo Analisa**

United States of America

**Donnez Jacques**

Belgium

**Dorsett Martin Wanda A**

American Samoa

**Downs Stephen**

United States of America

**Dursun Polat**

Turkey

**Eckstein Niels**

Germany

**Emoto Makoto**

Japan

**Er Tze Kiong**

Taiwan

**Ercan Cihangir Mutlu**

Turkey

**Evans Aco**

Ireland

**Fainzilber Mike**

Iran

**Falbo Angela**

Italy

**Feng Lingda**

China

**Ferrari Renata**

Brazil

**Fiorillo Lucia**

Italy

**Folgueira Maria Aparecida A Koike**

Brazil

**Foster Warren**

Canada

**Furlong Fiona**

United Kingdom

**Garcia Angel**

Spain

**Garcia Kara**

United States of America

**Geller Melissa**

United States of America

**Genazzani Alessandro**

Italy

**Georgopoulos Neoklis**

Greece

**Ghezzi Fabio**

Italy

**Goel Poonam**

India

**Gokce Cumali**

Turkey

**Gomez Raul**

Spain

**Grabowski Andrzej**

Poland

**Gu Jianren**

China

**Guedes Alexis**

Brazil

**Gungor Tayfun**

Turkey

**Guo Hua**

United States of America

**Gupta Digant**

United States of America

**Han Gencheng**

China

**Hashizume Kazuyoshi**

Japan

**Hawkridge Adam**

United States of America

**Hayashi Masahiro**

United States of America

**Hiraike Osamu**

Japan

**Hohenstein Peter**

United Kingdom

**Homburg Roy**

Israel

**Hovatta Outi**

Sweden

**Hsu Ming-I**

Taiwan

**Hua Jinlian**

China

**Huang HeFeng**

China

**Huhtinen Kaisa**

Finland

**Humaidan Peter**

Denmark

**Huntsman David**

Canada

**Iavazzo Christos**

Greece

**Irving-Rodgers Helen**

Australia

**Isidoro Ciro**

Italy

**Jana Barbara**

Poland

**Jiang Zhongli**

China

**Jiao Ze-Xu**

United States of America

**Jindal Sangita**

United States of America

**Johnson Joshua**

United States of America

**Kakar Sham**

United States of America

**Kantara Carla**

United States of America

**Kar Sujata**

India

**Karpf Adam**

United States of America

**Karuppaiyah Selvendiran**

United States of America

**Katabuchi Hidetaka**

Japan

**Katiyar Santosh**

United States of America

**Kenigsberg Shlomit**

Canada

**Kim Won Ho**

South Korea

**Kim Yun Hwan**

South Korea

**Kim Tae Joong**

South Korea

**Kiss-Toth Endre**

United Kingdom

**Knapp Pawel**

Poland

**Kobayashi Hiroshi**

Japan

**Koumarianou Anna**

Greece

**Krause Diane**

United States of America

**Kreeger Pamela**

United States of America

**Krzysztof Szyllo**

Poland

**Kucukgoz Gulec Umran**

Turkey

**Kumaramanickavel Govindasamy**

India

**Kurihara Hiroaki**

Japan

**Kushnir Vitaly**

United States of America

**Kyritsis Athanassios**

Greece

**La Sala Giovanni Battista**

Italy

**Le Tien**

Canada

**Leader Arthur**

Canada

**Ledger William**

Australia

**Lee Keun Ho**

South Korea

**Levy Tally**

Israel

**Li Xuelian**

China

**Lim Carol**

United States of America

**Lipay Monica**

Brazil

**Liu Ping**

China

**Ma Jinfeng**

China

**Machado Santelli Glaucia M**

Brazil

**Maggio da Fonseca Angela**

Brazil

**Mahner Sven**

Germany

**Mandato Vincenzo Dario**

Italy

**Mandel (molinas) Nil**

Turkey

**Mariani Luciano**

Italy

**Markman Maurie**

United States of America

**Markowska Janina**

Poland

**Masters Seth**

Australia

**Mateizel Ileana**

Belgium

**Matos Maria Helena**

Brazil

**Mayer Barbara**

Germany

**Meng Ryan**

China

**Mes-Masson Anne-Marie**

Canada

**Messinis Ioannis**

Greece

**Minegishi Takashi**

Japan

**Mitchell Gillian**

Australia

**Miyano Takashi**

Japan

**Moncef Benkhalifa**

France

**Moreno Barb**

Italy

**Morgante Giuseppe**

Italy

**Murphy Bruce**

Canada

**Muscogiuri Giovanna**

Italy

**Nakamura Yasuhiko**

Japan

**Nam Joo-Hyun**

South Korea

**Nassir Mani**

Germany

**Ng Ernest**

Hong Kong

**Nicoli Alessia**

Italy

**Nilsson Eric**

United States of America

**Ohmichi Masahide**

Japan

**Okada Kiyoshi**

Japan

**Oprea-Ilies Gabriela M**

United States of America

**Orio Francesco**

Italy

**Orisaka Makoto**

Japan

**Orvieto Raoul**

Israel

**Osuga Yutaka**

Japan

**Öztürk Turhan Nilgün**

Turkey

**Pados George**

Greece

**Palanimuthu Ponnusamy Moorthy**

United States of America

**Palmer Julia**

United Kingdom

**Palomba Stefano**

Italy

**Patankar Manish**

United States of America

**Patel Divya**

United States of America

**Pathy Sushmita**

India

**Paz Filho Gilberto**

Australia

**Pearl Michael**

United States of America

**Pejovic Tanja**

United States of America

**Pelosi Emanuele**

United States of America

**Peng Chun**

Canada

**Penzes Peter**

United States of America

**Piltonen Terhi**

Finland

**Pocard Marc**

France

**Porpora Maria Grazia**

Italy

**Poveda Andres**

Spain

**Rasekh Jahromi Athar**

Iran

**Ratajczak Mariusz**

United States of America

**Reiter Russel**

United States of America

**Richard Francois**

Canada

**Richards JoAnne**

United States of America

**Rivkin Saul**

United States of America

**Robert Claude**

Canada

**Rodgers Ray**

Australia

**Rodriguez Ana Beatriz**

Spain

**Rose Peter**

United States of America

**Roy Debarshi**

United States of America

**Rueda Bo**

United States of America

**Ruggeri Rosaria**

Italy

**Sairam M Ram**

Canada

**Sakumoto Ryosuke**

Japan

**Salih Sana**

United States of America

**Salvetti Natalia**

Argentina

**Samant Rajeev**

United States of America

**Sanada Sakiko**

Japan

**Saraga-Babic Mirna**

Croatia

**Sawicki Wlodzimierz**

Poland

**Sayki Arslan Muyesser**

Turkey

**Scalici Jennifer**

United States of America

**Schattman Glenn**

United States of America

**Schouten Philip**

Netherlands

**Schultz Matthew**

United States of America

**Sellappan Selvaraju**

India

**Seow Kok-Min**

Taiwan

**Shimada Masayuki**

Japan

**Shoji Tadahiro**

Japan

**Shu Yimin**

United States of America

**Silambanan Santhi**

India

**Singh Ajay**

United States of America

**Singh Shailesh**

United States of America

**Smith Peter**

New Zealand

**Smith Steven C**

Swaziland

**Smitz Johan**

Belgium

**Songsasen Nucharin**

United States of America

**Sonoda Kenzo**

Japan

**Sowter Heidi**

United Kingdom

**Speicher David**

United States of America

**Srivastava Rakesh**

United States of America

**Suganuma Nobuhiko**

Japan

**Sugino Norihiro**

Japan

**Sugiyama Toru**

Japan

**Sui Guangchao**

United States of America

**Sun Qing-Yuan**

China

**Sun Ying**

United Kingdom

**Tagliaferri Pierosandro**

Italy

**Tanos Vasilios**

Cyprus

**Telleria Carlos**

United States of America

**Teruyoshi Saito**

Japan

**Tsai Eing-Mei**

Taiwan

**Tsang Benjamin**

Canada

**Tukiendorf Andrzej**

Poland

**Tüttelmann Frank**

Germany

**Uccella Stefano**

Italy

**Vaclavikova Radka**

Czech Republic

**Vanderhyden Barbara**

Canada

**Vincenzo De Leo**

Italy

**Virant-Klun Irma**

Slovenia

**Wang Junjie**

China

**Wang Qi**

Canada

**Wang Qi**

United States of America

**Wheeler Stephanie B**

Austria

**Wiest Jonathan**

United States of America

**Wojakowski Wojciech**

Poland

**Wong Alice**

Hong Kong

**Wong P C**

Singapore

**Xue Kai**

China

**Yavuzcan Ali**

Turkey

**Yazbek Joseph**

United Kingdom

**Yokoyama Yoshihito**

Japan

**Yoshida Junji**

Japan

**Yoshino Kiyoshi**

Japan

**Zeillinger Robert**

Austria

**Zeimet Alain**

Austria

**Zhang Xia**

United States of America

**Zullo Fulvio**

Italy

